# Engineered *Pseudomonas aeruginosa* phages with quorum-quenching enzyme or depolymerase for inhibition of biofilm formation

**DOI:** 10.3389/fmicb.2025.1752980

**Published:** 2026-01-13

**Authors:** Xi Luo, Siyun Wang, Yongqing Yang, Ruyue Gao, Shengjian Yuan, Jialin Yu, Dong Liu, Xin Tan

**Affiliations:** 1 Department of Pediatrics, Affiliated Hospital of Zunyi Medical University (Guizhou Children's Hospital), Zunyi, Guizhou, China; 2 State Key Laboratory of Quantitative Synthetic Biology, Shenzhen Institute of Synthetic Biology, Shenzhen Institutes of Advanced Technology, Chinese Academy of Sciences, Shenzhen, China; 3 Department of Neonatology, Children’s Hospital of Chongqing Medical University, Chongqing Key Laboratory of Pediatrics, Chongqing, China; 4 Department of Neonatology, Shenzhen People’s Hospital (The Second Clinical Medical College, Jinan University; The First Affiliated Hospital, Southern University of Science and Technology), Shenzhen, China

**Keywords:** biofilm, CRISPR-Cas9, phage engineering, *Pseudomonas aeruginosa*, quorum-quenching lactonase, depolymerase

## Abstract

*Pseudomonas aeruginosa* is a major cause of healthcare-associated infections and a significant threat to global health, primarily due to its ability to form biofilms that protect it from host immune responses and block antibiotic efficacy. While bacteriophages (phages) are emerging as potential antimicrobial agents, their effectiveness is often limited by these bacterial biofilms. This study aimed to enhance the biofilm-disrupting capabilities of phages through genetic engineering. First, we validated the *in vitro* biofilm-inhibitory effects of two enzymes: the quorum-quenching lactonase (*Aiia*) and a phage-derived depolymerase (*DP*). To demonstrate their potential, we then used CRISPR-Cas9 to engineer the *P. aeruginosa* phage PaGZ-1 to express these biofilm-disrupting genes. The resulting engineered phages demonstrated superior inhibition of biofilm formation compared to the wild-type phage. Notably, the PaGZ-1-Aiia variant showed significant promise in both inhibiting biofilm formation and disrupting established biofilms. Our findings provide a straightforward method for introducing exogenous genes into non-model *P. aeruginosa* phage genomes, offering a novel and potentially effective strategy for combating drug-resistant, biofilm-forming infections.

## 1 Introduction

*Pseudomonas aeruginosa* (*P. aeruginosa*) is a significant cause of healthcare-associated infections, particularly severe in intensive care units (Fernández-Barat et al., 2017), whose threat to human health is not only localized but global (Qin et al., 2022). In many populations, infection with *P. aeruginosa* is associated with high morbidity and mortality, including among individuals with ventilator-associated pneumonia, chronic obstructive pulmonary disease (COPD), or cystic fibrosis (CF) (Ding et al., 2022; Oluyombo et al., 2019; Eklöf et al., 2020). It was classified as a high-priority pathogen on the World Health Organization’s (WHO) 2024 priority list of bacterial pathogens, highlighting the urgent need for research and development of new antibiotics (World Health Organization, 2024).

Biofilms formed by *P. aeruginosa* are well-known for being robust three-dimensional structures created by bacterial communities on biological or non-biological surfaces. These structures consist mainly of extracellular polymers secreted by bacteria, including polysaccharides, phospholipids, proteins, and extracellular DNA (Ghafoor et al., 2011). Once established, these biofilms enhance bacterial environmental adaptability, allowing them to evade host immune responses and block antibiotic penetration. This capability supports colonization and long-term persistence, leading to highly resistant chronic infections (Moradali et al., 2017), and presents a significant medical challenge. Therefore, eliminating *P. aeruginosa* and its biofilms is crucial.

Bacteriophages (phages), the most abundant viruses on Earth, are viruses that specifically infect bacteria. Unaffected by antibiotic resistance, they offer a biological treatment approach for drug-resistant bacteria. Due to their high host specificity and narrow antibacterial spectrum, phage preparations often require individualization, which led to their temporary replacement by antibiotics historically. However, with the rise of clinically relevant drug-resistant bacteria, phage therapy has regained attention. Crucially, phages not only replicate within and lyse their bacterial hosts, but certain phages produce depolymerases that degrade the extracellular polymeric substances (EPS) matrix, enhancing biofilm penetration (Lu and Collins, 2007). Nevertheless, naturally isolating a phage that exhibits specificity for target bacteria while simultaneously expressing relevant EPS-degrading enzymes is remains a significant challenge, often requiring laborious and low-throughput screening. Thus, engineering potent bactericidal phages to carry depolymerases is a more feasible approach.

However, a major hurdle in translating this approach lies in the lack of well-characterized model phages and the limited genomic understanding of native *P. aeruginosa* phages. Previous studies have engineered model phages to combat biofilms: T7 was armed with biofilm-disrupting genes (e.g., adhesin-degrading enzyme (*DspB*) (Lu and Collins, 2007), quorum-quenching lactonase (*Aiia*) (Pei et al., 2014), antimicrobial peptides (*AMPs*) (Lemon et al., 2019)), while M13 (a non-lytic *Escherichia coli* phage) was engineered as a targeted delivery vehicle for lytic phages against *P. aeruginosa* biofilms (Sun et al., 2022). These studies highlight a critical limitation: the engineered phages themselves (e.g., T7, M13) are not inherent anti-*P. aeruginosa* agents. Their activity is either confined to their native host bacteria or, as in the case of M13, dependent on a complex, multi-component system. Consequently, there exists a lack of a simple, standalone engineered phage that can directly and effectively target *P. aeruginosa* and its biofilms.

We therefore hypothesized that engineering a non-model *P. aeruginosa* phage to constitutively express a biofilm-degrading enzyme would yield a superior anti-biofilm agent. To test this hypothesis, this study engineered the non-model *P. aeruginosa* phage PaGZ-1, enhancing its bactericidal and biofilm-removal efficacy via expression of biofilm-disrupting genes. We first identified candidate genes and validated their encoded proteins’ ability to inhibit biofilm formation and degrade preformed biofilms *in vitro*. Using a CRISPR/Cas9-based strategy, we then identified an optimal genomic locus for transgene integration by inserting a monomeric red fluorescent protein (*mRFP*) reporter at various positions. Following confirmation of the optimal insertion site, we constructed recombinant phages expressing selected therapeutic genes and evaluated their anti-biofilm activity against *P. aeruginosa* PAO1.

## 2 Materials and methods

### 2.1 Bacterial strains, phages, plasmids and culture conditions

#### 2.1.1 Bacterial strains and culture

The *P. aeruginosa* strains used in this study included the reference strains PAO1 and PA14, kindly provided by Dr. Fan Jin’s laboratory at the Shenzhen Institute of Synthetic Biology, as well as 100 clinical isolates. These clinical isolates were obtained from sputum samples at Shenzhen University General Hospital between January 2020 and December 2022. These isolates were characterized by multi-locus sequence typing (MLST) according to the standard scheme for *P. aeruginosa* (amplification and sequencing of seven housekeeping genes: *acsA*, *aroE*, *guaA*, *mutL*, *nuoD*, *ppsA*, and *trpE*). Allele and sequence type (ST) assignments were determined using the *P. aeruginosa* MLST database.^1^

All bacterial strains were routinely cultured in Luria–Bertani (LB) broth or on LB agar (1.5% agar) at 37 °C. For specific procedures (e.g., phage infection, transformation), bacteria were grown to the appropriate optical density as detailed in subsequent sections.

#### 2.1.2 Phages and propagation

The lytic phage PaGZ-1, a member of the *Pakpunavirus* genus (family *Myoviridae*), was originally isolated from fresh water in Guangdong province, China. Its complete genome (GenBank accession MH791399) is 93,975 bp in size, and it has been previously characterized to exhibit efficient lytic activity against various *P. aeruginosa* hosts (Wen et al., 2020). PaGZ-1 was selected as the engineering platform following a systematic screen. To identify phages amenable to CRISPR-Cas9-mediated genome editing, we tested five representative *P. aeruginosa* phages from distinct genera (Yualikevirus, Bruynoghevirus, Pakpunavirus, Phikmvvirus, Pbunavirus). Edibility was assessed by attempting to delete four distinct non-essential genes in each phage. PaGZ-1 was the only candidate that supported successful gene deletion and was therefore chosen for all subsequent engineering. Phage propagation and titration: Phage PaGZ-1 was propagated on *P. aeruginosa* PAO1 as the host strain. Phage titers (plaque-forming units, PFU/mL) were determined using the standard double-agar overlay plaque assay on LB agar plates, followed by incubation at 37 °C for 18–24 h.

#### 2.1.3 Plasmids and construction

The type II CRISPR-Cas9 system from *Streptococcus pyogenes* was employed for phage genome editing (Chen et al., 2018). The plasmids pCasPA (Addgene #113347) and pACRISPR (Addgene #113348) were obtained from Dr. Fan Jin’s laboratory.

pCasPA encodes Cas9, the λ-Red recombination system, a tetracycline resistance marker, and the SacB gene. It was electroporated into PAO1 to generate the recipient strain PAO1 (pCasPA). This strain was maintained in LB medium supplemented with 50 μg/mL tetracycline.

pACRISPR was used as the donor plasmid for homologous recombination. Spacer sequences (20-nt), homology arms (350-500 bp) and the candidate gene (*mRFP*, *Aiia*, or depolymerase gene) driven by the constitutive promoter J23100 were assembled into this backbone via overlap extension PCR and cloned using the ClonExpress II One Step Cloning Kit (Vazyme, C112-01).

Recombinant plasmid transformation and selection: The constructed pACRISPR-derived plasmids were electroporated into the PAO1 (pCasPA) strain. Transformants were selected on LB agar plates (1.5% agar) supplemented with 100 μg/mL tetracycline and 150 μg/mL carbenicillin, followed by incubation at 37 °C for 18–24 h.

### 2.2 Cloning, expression, and purification of *Aiia* and *DP* proteins

**Figure 1:**
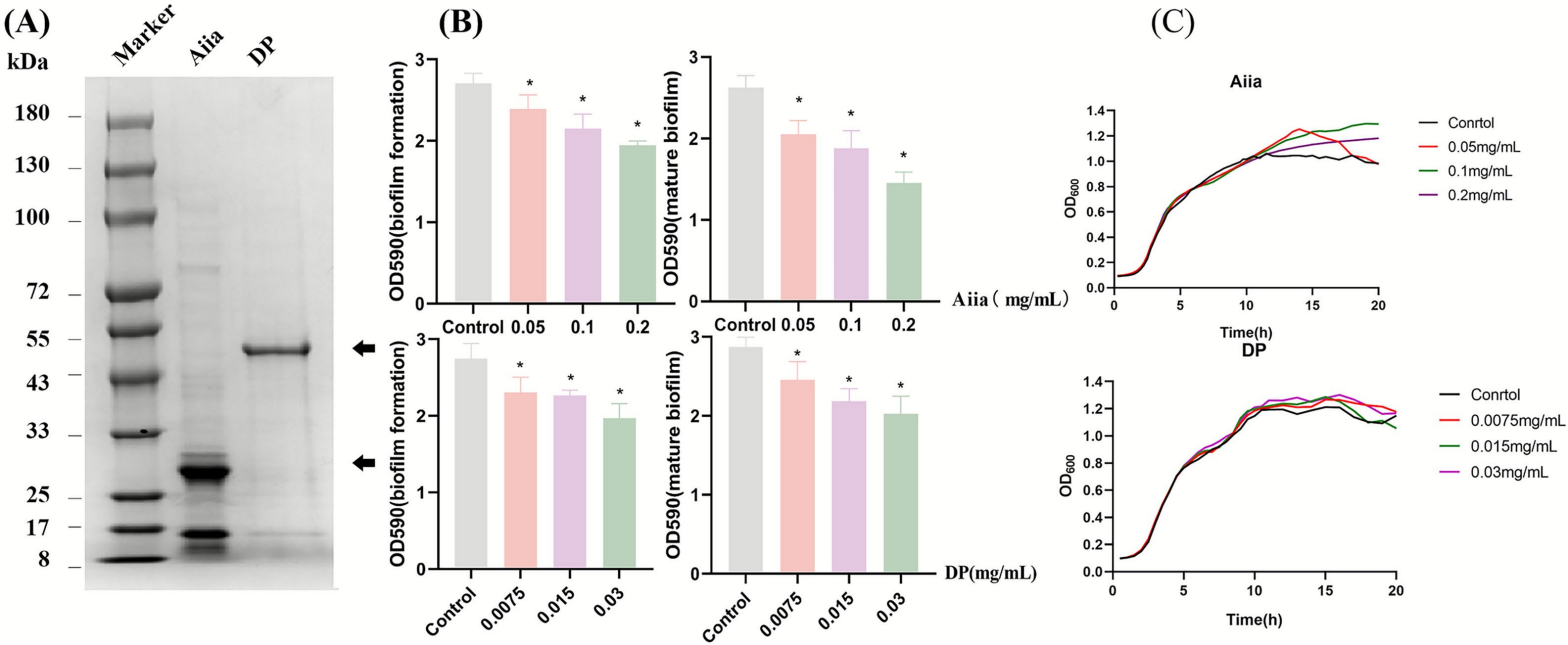
Purification and functional validation of *Aiia* and *DP* proteins. **(A)** Purification of recombinant proteins analyzed by 12% SDS-PAGE. His-tagged *Aiia* and *DP* were expressed in *E. coli* upon IPTG induction and purified by Ni–NTA affinity chromatography (elution with 500 mM imidazole). The observed molecular weights correspond to the predicted sizes of *Aiia* (~29 kDa) and *DP* (~49 kDa). **(B)** Anti-biofilm activity of purified proteins. (Left) Inhibition of biofilm formation: PAO1 was co-incubated with the indicated concentrations of *Aiia* or *DP*. (Right) Disruption of pre-formed mature biofilms: established biofilms were treated with proteins. Residual biofilm biomass was quantified by crystal violet staining. Data are mean ± SD of three biological replicates. *p < 0.05 (one-way ANOVA with Dunnett’s post-hoc test compared to the untreated control). **(C)** Bacterial growth curves. PAO1 was cultured in LB broth with or without (Control) the addition of *Aiia* or *DP* proteins. Growth (OD_600_) was monitored over time. Data are representative of three independent experiments.

The *Aiia* gene (GenBank: MN227141) and *DP* gene (GenBank: MF788075) were synthesized by Beijing Liuhe BGI Co., Ltd. (Beijing). Primers sequences for *Aiia* and *DP* genes amplification were listed in Supplementary Table S1. The target genes were expressed in a pET vector as N-terminal fusions to a SUMO-His tag. The SUMO moiety was included to enhance protein solubility, while the His tag enabled one-step purification using Ni–NTA affinity chromatography. The recombinant plasmids pGEX-6p-3-Aiia and pGEX-6p-3-*DP* were separately transformed into *E. coli* BL21 (DE3) (TransGen Biotech, Beijing). Based on optimization of IPTG concentration and temperature, the final expressions were performed at 16 °C for 16 h with 0.5 mM IPTG for pGEX-6p-3-Aiia and 1 mM IPTG for pGEX-6p-3-DP. These conditions were found to maximize soluble yield while minimizing inclusion body formation for each respective construct. Cells were harvested and disrupted by sonication. The purified fusion proteins were used directly in all subsequent experiments. The functional assays presented in this study (as shown in Figures 1B,C, etc.) were conducted with the SUMO-fused proteins and demonstrate that the prepared protein samples possess the intended biological activity. Protein molecular weights were confirmed by FuturePAGE™ 4–12% SDS-PAGE (ACE Biotechnology, Hunan) and stained with BeyoBlue™ Coomassie Blue (Beyotime Biotechnology, Shanghai). Protein concentrations were quantified using the Bradford assay (Thermo Scientific™, Massachusetts) with bovine serum albumin (BSA) standards. Purified proteins were dissolved in PBS (pH 8.0) and stored at −80 °C.

### 2.3 Construction of engineered phage via CRISPR-Cas9 editing

Electrocompetent *P. aeruginosa* PAO1 cells were prepared as previously described (Choi et al., 2006). Briefly, a mid-log phase culture (OD₆₀₀ ~ 0.6) was harvested, washed twice with ice-cold 300 mM sucrose, and resuspended in the same solution. To generate the editing host strain, plasmid pCasPA (encoding Cas9 and the *λ*-Red recombination proteins) was first electroporated into PAO1. Transformants were selected on LB agar containing 50 μg/mL tetracycline, yielding strain PAO1 (pCasPA). Subsequently, the donor plasmid pACRISPR—which carries the sgRNA expression cassette, the homology arms, and the engineered gene insert (e.g., *aiiA*)—was electroporated into PAO1 (pCasPA). Double transformants were selected on plates supplemented with both tetracycline (50 μg/mL) and carbenicillin (150 μg/mL). The resulting strain, designated PAO1 (pCasPA, pACRISPR), served as the host for phage genome editing.

Rationale for three-cycle phage amplification: Wild-type PaGZ-1 phage was then used to infect this engineered host strain at a low multiplicity of infection (MOI < 0.1). During lytic replication in this host, the Cas9/sgRNA complex targets and cleaves the wild-type phage genome at the intended site. Concurrently, the *λ*-Red system promotes homologous recombination between the phage genome and the donor template on pACRISPR, thereby incorporating the engineered gene. This infection and recombination process was iterated for three consecutive cycles by repeatedly harvesting the lysate and using it to re-infect fresh PAO1 (pCasPA, pACRISPR). This step is critical to enrich the population of recombinant phages over the wild-type background.

Following the third amplification cycle, the lysate was serially diluted and subjected to plaque assay on plasmid-free PAO1. Randomly selected single plaques were picked and initially screened by PCR using insertion-flanking primers (1-insert-test-F/R or 2-insert-test-F/R). Putative recombinant phages were purified through three additional rounds of plaque isolation on plasmid-free PAO1 to ensure clonal purity. Final validation was performed by colony PCR and Sanger sequencing of the modified genomic region. All primer sequences are listed in Supplementary Table S1.

### 2.4 Phage characterization assay

Phage host range determination: Bacterial lawns were prepared by mixing 200 μL of overnight cultures with 4 mL molten 0.75% LB agarose, then overlaid on LB agar plates. The phage stock at a concentration of 1 × 10^11 PFU/mL was serially diluted in 10-fold steps (10^^−1^ to 10^^−8^). Aliquots (10 μL) of each dilution were spotted onto the bacterial lawns. Plates were incubated at 37 °C for 18–24 h, and Efficiency of Plating (EOP) was assessed.

To compare fundamental properties between engineered and wild-type (WT) phages, we assessed plaque morphology, conducted one-step growth assay, monitored bacterial growth kinetics, and examined virion morphology and size by transmission electron microscopy (TEM). For all assays requiring phage amplification, PAO1 in the logarithmic growth phase (OD₆₀₀ = 0.5–0.6) was used as the host.

Plaque morphology of PaGZ-1 and engineered phages was examined on PAO1 using double-agar overlay assays. Briefly, 200 μL PAO1 culture and 10 μL serially diluted phage suspension were mixed with 5 mL molten 0.75% LB agarose, overlaid on pre-warmed LB agar plates, and incubated at 37 °C for 18 h.

Bacterial growth kinetics were monitored as reported (Yuan et al., 2022). Log-phase PAO1 was infected with phages at MOI 0.00001. Then, 200 μL mixture were dispensed into 96-well plates with triplicate wells per group. LB medium alone and uninfected PAO1 served as negative and positive controls, respectively. OD600 was recorded every 30 min for 24 h at 37 °C with continuous shaking using a microplate reader.

One-step growth curves were performed as previously described (Yuan et al., 2022). A 10 mL culture of PAO1 was centrifuged (7,000×g, 3 min), resuspended in 1 mL of LB, and infected with phage at MOI 0.01 for 5 min at 37 °C to allow adsorption. Fresh LB (9 mL) was added and the cultured was incubated at 37 °C with 220 rpm shaking. Aliquots (10 μL) were collected at 5–10 min intervals for 50 min. The number of plaque-forming units (PFU) per mL was determined by spot titration. The burst size, defined as the number of progeny phage released per infected cell, was calculated as the ratio of the final PFU titer after the rise period to the initial PFU titer of the infecting phage.

For visualization and size measurement of phage particles, high-titer lysates of PaGZ-1 and its engineered variants were prepared. Briefly, phages were amplified in PAO1 to obtain a high yield. The lysates were purified by polyethylene glycol (PEG) precipitation and ultracentrifugation. Purified phage particles were resuspended in SM buffer.

For TEM imaging, 10 μL of the purified phage suspension was applied onto a carbon-coated copper grid for 1 min, and the excess liquid was blotted away. The grid was then negatively stained with 2% (w/v) uranyl acetate for 30 s. After air-drying, samples were imaged using a Transmission Electron Microscope (Tecnai 12, Philips Company) operated at an accelerating voltage of 80 kV. The dimensions (capsid diameter and tail length) of at least 20 individual virions per sample were measured using image analysis software.

### 2.5 Quantification of mRFP fluorescence

Log-phase PAO1 was diluted to 10^5^ CFU/mL, and infected with phages at MOIs of 1, 0.1,0.01, 0.001,0.0001. Aliquots (200 μL) of each mixture were dispensed into 96-well microplates with triplicate wells per condition. LB medium alone and uninfected PAO1 served as negative and positive controls, respectively. Plates were incubated at 37 °C with continuous shaking. mRFP fluorescence intensity (excitation 560 nm/emission 640 nm) was measured every 10 min for 240 min using a fluorescence microplate reader (qTOWERiris touch, analytik jena).

### 2.6 qRT-PCR

Gene expression analysis was conducted as follows (Lemon et al., 2019): Mid-log phase PAO1 cultures were infected with phage at an MOI of 1 for 30 min; after centrifugation, total RNA was extracted from the pellet (RNAprep Pure Cell/Bacteria Kit, TIANGEN) and reverse-transcribed into cDNA (TB Green Premix Ex Taq™, TaKaRa); qRT-PCR was then performed using a One Step TB Green PrimeScript™ PLUS RT-PCR Kit (RR096A, TaKaRa) on a StepOnePlus™ system (Applied Biosystems, Thermo Fisher). The relative expression levels of target genes were calculated using the comparative ΔΔCt method, with the phage gene *DprA* serving as the reference. *DprA* was predicted to be expressed at an early stage of PaGZ-1 infection. All primers used in this study are listed in Supplementary Table S1.

### 2.7 Biofilm formation and antibiofilm assays

For biofilm formation inhibition and mature biofilm degradation assays, mid-log phase PAO1 was used. For the biofilm formation inhibition assay, bacteria were mixed with purified proteins (*Aiia*: 0.05, 0.1, 0.2 mg/mL; *DP*: 0.0075, 0.015, 0.03 mg/mL; PBS as control) or phages (MOI= 0.00001) in 96-well microtiter plates (total volume 200uL). Protein-treated groups were incubated at 30 °C for 18 h and phage-treated groups for 12 h. For the mature biofilm degradation assay, PAO1 was first cultured in microtiter plates for 36 h to establish biofilms. After discarding spent media, fresh LB containing proteins (at the same concentrations as above) or phages (2 × 10^6^ PFU/mL) was added (200 μL/well) and incubated for 6 h at 30 °C.

Post-treatment, the supernatant was aspirated, and the wells were washed twice with 1× PBS to remove planktonic bacteria. The plates were then air-dried at 37 °C for 15 min. Subsequently, the adherent biofilms were stained with 0.1% crystal violet (Solarbio, G1059) for 20 min at room temperature. Excess dye was removed by washing three times with PBS, and the bound dye was eluted with 200ul 95% ethanol (30 min, RT) for quantification at OD₅₉₀ (BioTek Synergy H1). Three independent experiments were performed (six technical replicates each). Contaminated wells were excluded from analysis.

### 2.8 Microscopy techniques for biofilm imaging

For scanning electron microscopy (SEM) of biofilms, PAO1 was cultured in 24-well plates containing sterile glass coverslips at 37 °C with daily medium replacement. After 72 h, supernatants were gently removed and replaced with 1 mL phages suspensions (equivalent PFU) per well, followed by incubation at 30 °C for 6 h. Coverslips were washed twice with sterile PBS and fixed overnight at 4 °C in pre-chilled 2.5% glutaraldehyde. Samples were dehydrated through an ethanol series, critical-point dried, and sputter-coated with gold prior to imaging on a Tecnai 12 SEM (Philips).

For confocal laser scanning microscopy (CLSM), SYTO 9 and propidium iodide (PI) were mixed 1:1. Biofilm-coated coverslips were stained with 50 μL dye mixture for 20 min in the dark, gently rinsed with PBS, and mounted with 70% glycerol. Imaging was performed using a two-photon confocal microscope (Leica SP8 DIVE/Falcon, Germany) with argon laser excitation (488 nm). Z-stacks were acquired from the biofilm interface to the substratum. Three random fields per slide were analyzed, with viable bacteria fluorescing green and dead cells red.

### 2.9 Statistical analysis

Statistical analyses were performed using SPSS 25.0 (IBM) and GraphPad Prism 8 (San Diego, CA, USA). Data conforming to normal distribution and homogeneity of variance are expressed as mean ± standard deviation (X ± s). Comparisons between two groups were analyzed by unpaired Student’s t-test. For comparisons involving multiple experimental groups against a single control group, a one-way ANOVA was performed, followed by Dunnett’s post-hoc test. Image analysis utilized Image-Pro Plus 6.0 (Media Cybernetics). Statistical significance was defined as *p* < 0.05 for all tests.

## 3 Results

### 3.1 Recombinant *Aiia* lactonase and *DP* protein inhibited biofilm formation and disrupted mature biofilm

Plasmids containing the *Aiia* gene (747 bp) and *DP* gene (1,389 bp) were separately cloned downstream of SUMO Tags in the PGEX-6p-3 Vector (Supplementary Figure S1). Recombinant plasmids were transformed into *E. coli* BL21 (DE3). Following expression induction and affinity purification via their His-tags, SDS-PAGE analysis confirmed the successful purification of *Aiia* (29 kDa) and *DP* (49 kDa) proteins (Figure 1A).

The antibiofilm activity of *Aiia* and *DP* against PAO1 was validated *in vitro*. Both proteins not only inhibited biofilms formation, but also disrupted preformed mature biofilms in a dose-dependent manner (Figure 1B). This effect was not attributable to growth inhibition (Figure 1C). It should be noted that this activity was assessed under optimal conditions for the isolated proteins, which may differ from their expression and functional context within an engineered phage during infection. Nevertheless, the significant clearance capacity demonstrated their suitability as candidate genes for phage engineering.

### 3.2 Selection of phage PaGZ-1 for engineering and validation of insertion site

Based on the screening results described in Section 2.1.2, phage PaGZ-1 emerged as the only candidate that supported successful gene deletion, and was therefore selected for all subsequent engineering.

**Figure 2:**
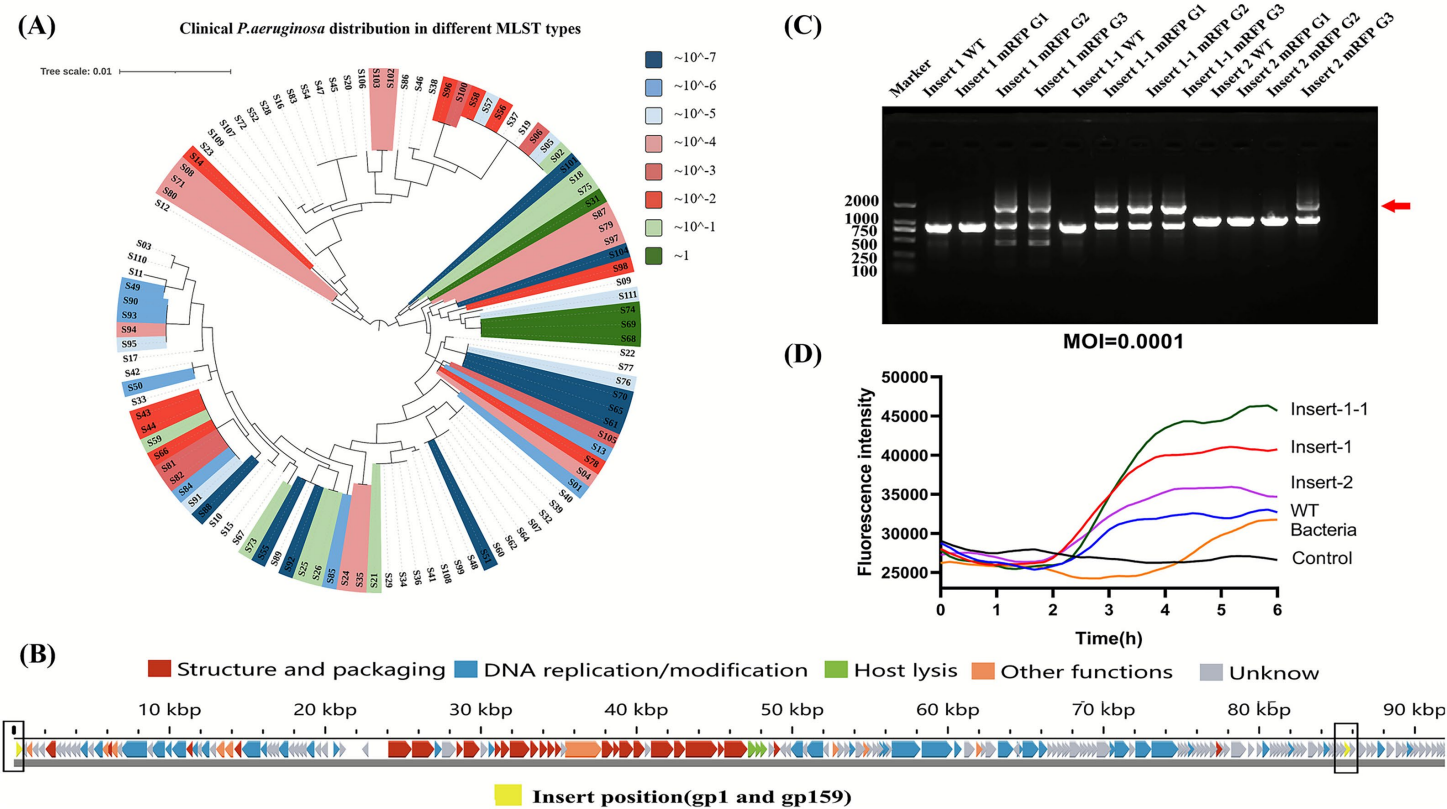
Selection of phage PaGZ-1 as engineering chassis and insertion site validation. **(A)** Multi-locus sequence typing (MLST) profile of 100 clinical *P. aeruginosa* isolates and the host range of wild-type PaGZ-1. PaGZ-1 lysed 61 of the tested strains (colors marking). Isolates are color-coded according to the efficiency of plating (EOP) value ranges, as defined in the key. Non-permissive hosts (EOP = 0) are shown without color. **(B)** Schematic of the PaGZ-1 genome (93,975 bp) and selected insertion sites. Open reading frames (ORFs) are colored by predicted functional modules. Three insertion sites (yellow, black-framed) within two non-essential genes (*gp1* and *gp159*) were chosen for the integration of an *mRFP* expression cassette. **(C)** PCR validation of *mRFP* insertion. Genomic DNA from wild-type (WT) phage and from progeny after one (G1), two (G2), or three (G3) rounds of amplification in the CRISPR-Cas9 editing host was analyzed with primers flanking the target sites. Successful recombination is indicated by the appearance of a larger amplicon (red arrow) compared to the WT band. **(D)** Fluorescence intensity of phage lysates carrying *mRFP* at the three different insertion sites. Lysates were normalized by plaque-forming unit (PFU) titer.

MLST of 100 clinical antibiotic-resistant isolates revealed 39 distinct sequence types. Host range analysis demonstrated PaGZ-1’s broad-spectrum lytic activity against these isolates. The efficiency of plating (EOP) was calculated relative to the standard host strain PAO1. PaGZ-1 productively infected 61 strains, with EOP values distributed across several orders of magnitude (Figure 2A).

We targeted four non-essential gene loci to preserve native phage functions; these sites were identified as hypothetical proteins through RAST-based genome annotation and NCBI BLASTP analysis, which confirmed the absence of sequences essential for phage replication, structural integrity, or packaging. The successful integration of the mRFP reporter at two loci—*gp1* (at two positions: insert_1 and insert_1–1) and *gp159* (insert_2)—is indicated by yellow markers within black outlining boxes in Figure 2B. Recombinant phages emerged after passages 2–3 of infection in PAO1 harboring the pCasPA and pACRISPR plasmids. PCR analysis of the lysates yielded two bands, with the longer band (marked by a red arrow) indicative of successful foreign gene insertion (Figure 2C) and were purified through single-plaque isolation. Fluorescence intensity analysis confirmed insert_1–1 as the optimal site for exogenous gene expression (Figure 2D).

### 3.3 Construction of engineered phage PaGZ-1-Aiia and PaGZ-1-DP

**Figure 3:**
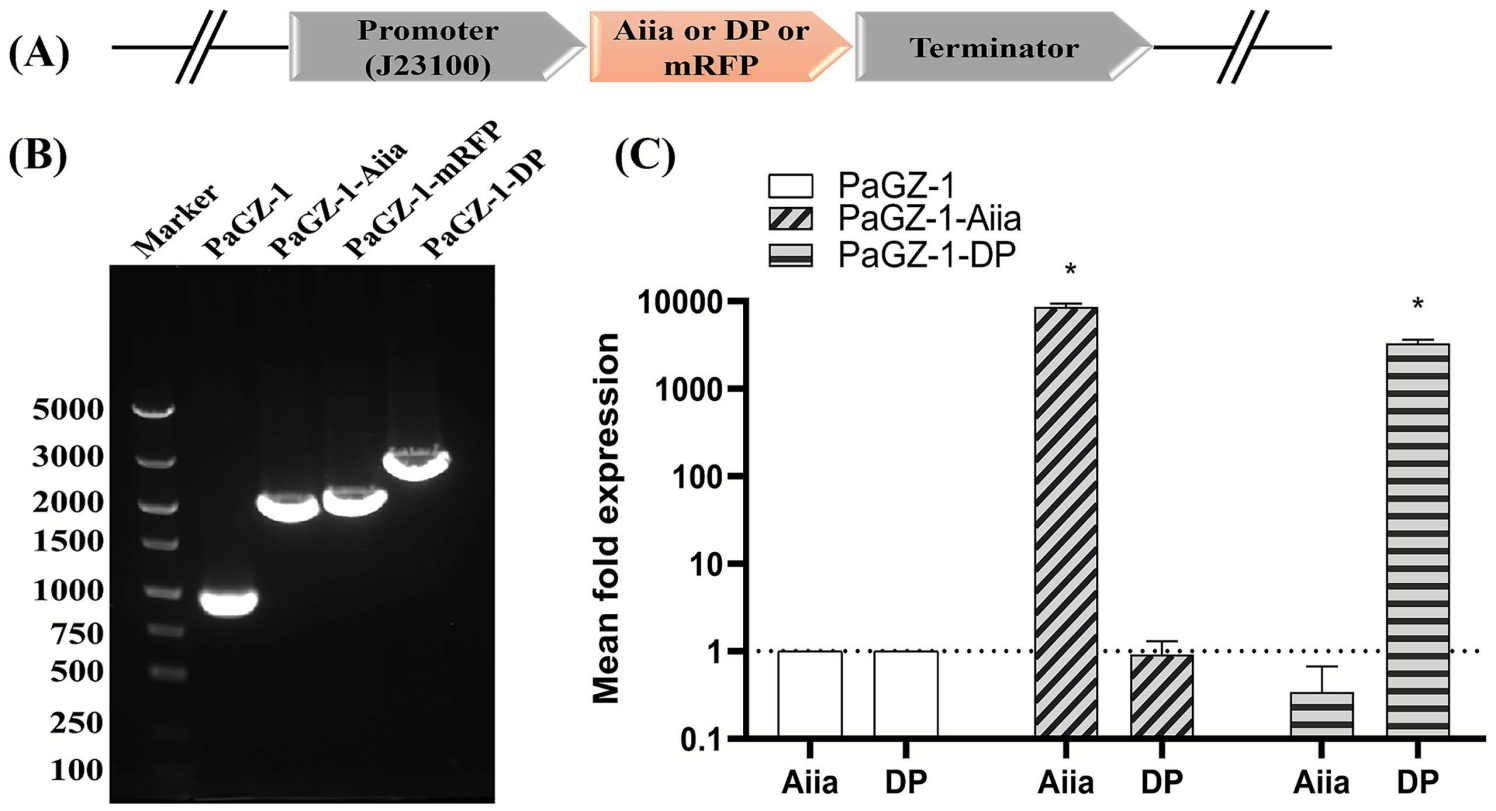
Construction and validation of engineered phages PaGZ-1-Aiia and PaGZ-1-DP. **(A)** Schematic of the CRISPR-Cas9-mediated insertion strategy at the *insert_1–1* locus. Expression cassettes containing a constitutive promoter (J23100), the coding sequence for *Aiia*, depolymerase (*DP*), or *mRFP*, and a terminator were integrated. The diagram is not drawn to scale. **(B)** PCR validation of genetic insertion. Genomic DNA from wild-type (WT) and engineered phages was amplified with primers flanking the *insert_1–1* locus. The observed amplicon sizes correspond to the WT (915 bp) and recombinant phages carrying *Aiia* (1967 bp), *DP* (2,608 bp), or *mRFP* (1948 bp) cassettes. **(C)** Quantitative reverse transcription PCR (qRT-PCR) analysis of engineered gene expression. *P. aeruginosa* PAO1 was infected with WT or engineered phages. Relative transcript levels of *Aiia* (in PaGZ-1-*Aiia*) and *DP* (in PaGZ-1-DP) were quantified at 30 min post-infection and normalized to the WT phage control. Data are mean ± SD of three biological replicates. *p < 0.05 (one-way ANOVA with Dunnett’s post-hoc test compared to WT).

Successful integration of the *Aiia* or *DP* expression cassettes (Figure 3A) was confirmed by Sanger sequencing. PCR amplification with primers 1-insert-test-F/R verified insertion at the insert_1–1 locus of PaGZ-1, yielding fragments of 915 bp (WT), 1967 bp (PaGZ-1-Aiia), 1948 bp (PaGZ-1-mRFP), and 2,608 bp (PaGZ-1-DP) (Figure 3B). All constructs were validated by Sanger sequencing. qRT-PCR analysis was performed to measure the expression of the introduced genes in the engineered phages relative to the parental phage PaGZ-1, at 30 min post-infection of PAO1. The results confirmed the specific expression of each gene in its respective engineered phage: the *Aiia* gene was highly expressed in phage PaGZ-1-Aiia (8,621-fold higher than in PaGZ-1), while the *DP* gene was highly expressed in phage PaGZ-1-DP (3,278-fold higher than in PaGZ-1) (Figure 3C).

### 3.4 Characterization of engineered phage PaGZ-1-Aiia and PaGZ-1-DP

**Figure 4:**
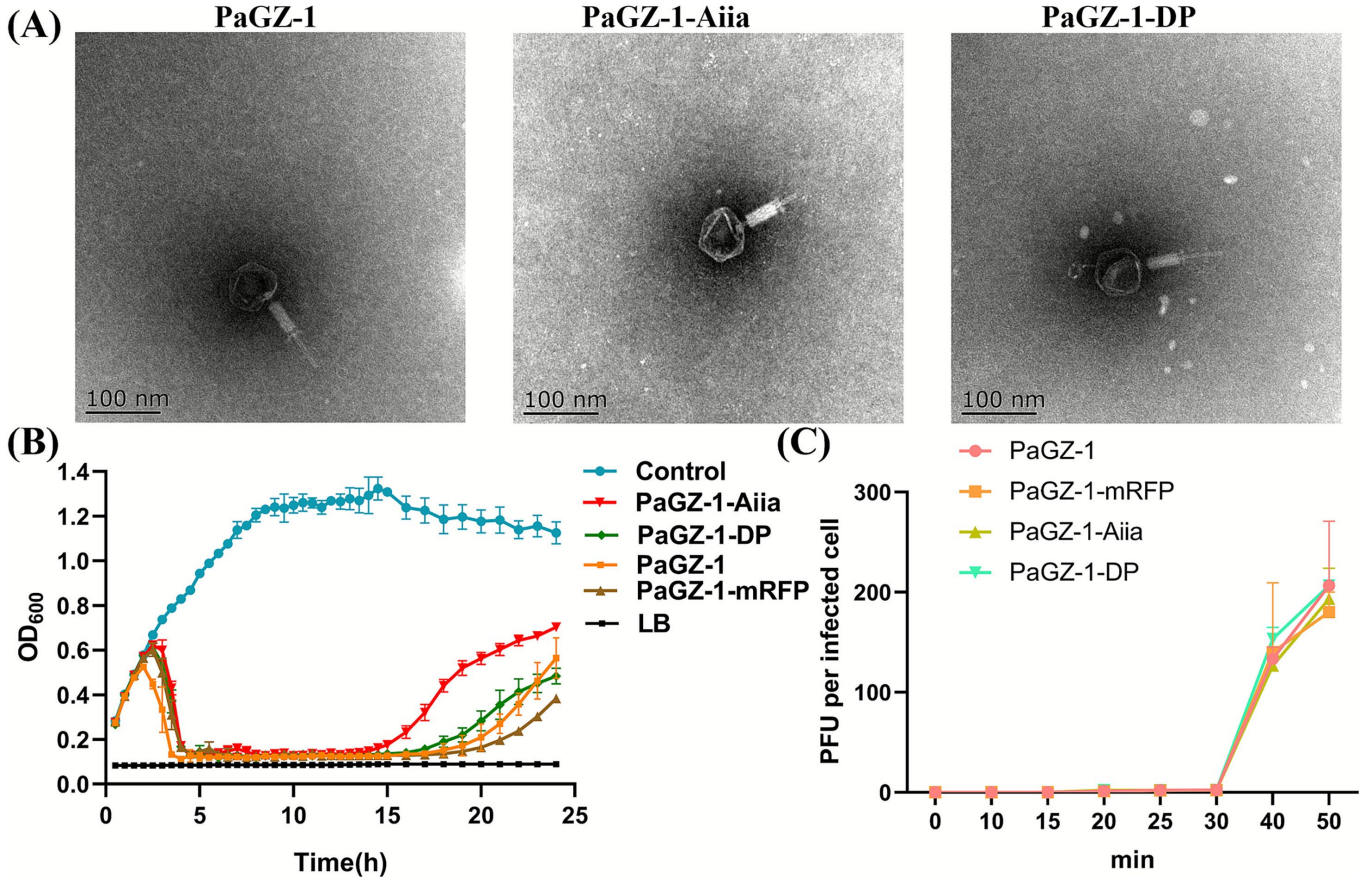
Characteristics of wild-type (WT) and engineered phages. **(A)** Representative TEM images of the wild-type phage PaGZ-1 and the engineered derivatives PaGZ-1-Aiia and PaGZ-1-DP. Average dimensions are indicated. Scale bar: 100 nm. **(B)** Growth inhibition of *P. aeruginosa* PAO1 by phage infection. Bacterial cultures were infected at a low multiplicity of infection (MOI = 1 × 10^−6^), and optical density (OD_600_) was monitored over time. Data are representative of three independent experiments. **(C)** One-step growth curves. PAO1 was infected with phages at an MOI of 0.01. Samples were taken at the indicated time points to determine the plaque-forming unit (PFU) titer. The burst size was calculated from the plateau phase. Data are mean ± SD of three biological replicates.

We characterized both engineered phages to assess potential deviations from wild-type (WT) PaGZ-1. TEM revealed near-identical morphologies for PaGZ-1-Aiia and PaGZ-1-DP compared to WT, with average dimensions of 61 nm (head width) × 64 nm (head length) and tail lengths of ~57 nm (Figure 4A). No significant structural differences were observed.

Growth inhibition assays at MOI 0.000001 demonstrated potent suppression of PAO1 by both WT and engineered phages for >15 h. Bacterial regrowth was most pronounced in cultures treated with PaGZ-1-AiiA. This regrowth could be attributed to the potential emergence of phage-resistant bacterial subpopulations, a phenomenon commonly observed during phage therapy (Figure 4B).

One-step growth curves showed identical 30-min latent periods for all phages, plateauing at 50 min with comparable burst sizes (81–91 progeny per infected cell; *p* > 0.05) (Figure 4C).

Plaque morphology analysis after 24–72 h incubation revealed that all phages produced translucent plaques with halos. The halo sizes were largely consistent across all phages, including PaGZ-1-DP, which did not exhibit any enlargement relative to the others (Supplementary Figure S2). Collectively, the insertion of *Aiia* or *DP* preserved the core biological properties of the phage.

### 3.5 Engineered phage PaGZ-1-Aiia and PaGZ-1-DP inhibits PAO1 biofilms

**Figure 5:**
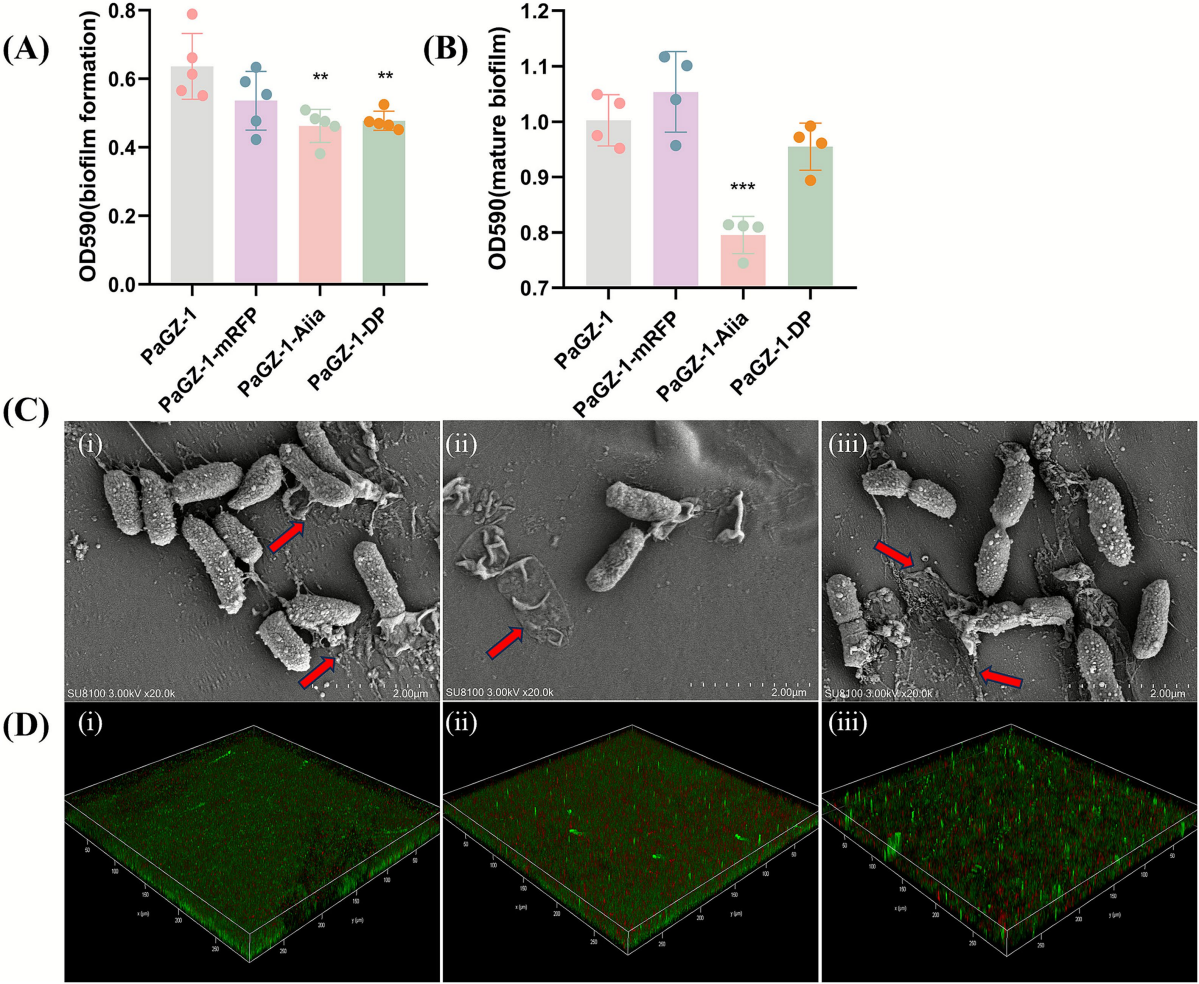
Inhibition and disruption of *P. aeruginosa* PAO1 biofilms by engineered phages. **(A)** Inhibition of new biofilm formation. Biofilm biomass was quantified by crystal violet staining after co-incubation of bacteria with wild-type PaGZ-1 or engineered phages at an MOI of 0.00001. Data are mean ± SD; **p < 0.01 (one-way ANOVA with Dunnett’s test compared to WT). **(B)** Disruption of pre-formed mature biofilms. Established (36 h) biofilms were treated with phages, and residual biomass was quantified. Data are mean ± SD; ***p < 0.001 (one-way ANOVA with Dunnett’s test compared to WT). **(C)** Representative scanning electron microscopy (SEM) images of mature biofilms after treatment with (i) WT PaGZ-1, (ii) PaGZ-1-AiiA, or (iii) PaGZ-1-DP. Red arrows indicate the filamentous extracellular polymeric substance (EPS) matrix. Scale bar, 2 μm. **(D)** Representative confocal laser scanning microscopy (CLSM) images of biofilms after treatment with (i) WT PaGZ-1, (ii) PaGZ-1-AiiA, or (iii) PaGZ-1-DP. Biofilms were stained with SYTO 9 (green, live cells) and propidium iodide (red, dead cells).

In biofilm inhibition assays at MOI 0.00001, both engineered phages significantly suppressed new biofilm formation (*p* < 0.01, crystal violet staining; Figure 5A). However, when disrupting pre-formed mature biofilms (36 h), a striking difference was observed: only PaGZ-1-AiiA caused a substantial reduction in biomass (*p* < 0.0001 compared to the wild-type phage), whereas PaGZ-1-DP did not perform significantly better than the wild-type phage (Figure 5B). It is important to note that crystal violet staining quantifies total biofilm biomass without differentiating between effects on bacterial viability and physical disruption of the extracellular matrix. Therefore, we subsequently used SEM and CLSM for direct visualization of biofilm architecture and to quantify live/dead cells.

SEM imaging (Figure 5C) revealed dense biofilms with abundant filamentous, network-like extracellular material—characteristic of dehydrated EPS—in the wild-type (WT) phage-treated groups. In contrast, treatment with PaGZ-1-Aiia led to a marked disruption of biofilm architecture, evident as a dispersion of the bacterial clusters, a reduction in EPS, and a decrease in overall cell density. This biofilm-disrupting effect was not observed in the PaGZ-1-DP groups.

CLSM quantified biofilm thickness (Figure 5D): WT phage (19.33 ± 3.11um) was significantly higher than PaGZ-1-Aiia phage (12.33 ± 0.44um, *P* < 0.05), and no statistical difference with PaGZ-1-DP phage (14.00 ± 1.33um). Dead/live cell ratios (red/green fluorescence) increased significantly in engineered phage groups: 0.17 ± 0.02 (*Aiia*) and 0.17 ± 0.04 (*DP*) vs. WT (0.06 ± 0.02; *p* < 0.05).

## 4 Discussion

Phages are natural agents that inhibit biofilms and demonstrate promising efficacy in both preventing and disrupting biofilm formation. Studies indicate that the antibiofilm activity of certain phages extends beyond the virions themselves, involving enzymes such as endolysins and depolymerases encoded in their genomes; these phages exhibit greater efficacy than those lacking such enzymes (Asma et al., 2022). Consequently, phages lacking genes encoding biofilm-degrading enzymes can be genetically engineered to express antibiofilm enzymes during infection (Peng and Chen, 2021). Current efforts to express exogenous genes primarily focuses on model phages (e.g., *E. coli* phages T7, T4, *λ*, and M13) (Pei et al., 2014; Sun et al., 2022; Pavoni et al., 2013; Tao et al., 2013). However, no model phage exists for *P. aeruginosa* and heterologous gene expression in non-model phages remains challenging due to limited genomic annotation.

Therefore, selecting suitable wild-type *P. aeruginosa* phages for genetic engineering warrants careful consideration. Based on our laboratory’s *Pseudomonas* phage library, we screened seven diverse phages using CRISPR-Cas9 tools. Only phages of the genus *Pakpunavirus* supported successful editing. PaGZ-1, a member of this genus, exhibits a broad host range—lysing 61% (61/100) of clinical drug-resistant *P. aeruginosa* isolates—demonstrating potent lytic activity. A second challenge involves editing tool compatibility. Previous studies report that the jumbo phage ФKZ resists DNA-targeting immune systems via a proteinaceous “phage nucleus” shielding its replicating genome (Mendoza et al., 2020; Malone et al., 2020), necessitating CRISPR-Cas13a for editing (Guan et al., 2022). Unlike ФKZ, PaGZ-1 permitted direct CRISPR-Cas9-mediated gene deletions and insertions, establishing it as a candidate model phage for future research.

Following successful integration of exogenous genes into PaGZ-1, we confirmed transcript translation via direct mRFP fluorescence observation (Figure 2D) and validated the phage’s physiology was not adversely affected by insertion (Figure 4). This demonstrates PaGZ-1’s suitability as a flexible chassis for exogenous gene expression. Given CRISPR-Cas9’s dual utility for gene deletions, future work could delete non-essential loci (Yuan et al., 2022) prior to inserting therapeutic genes to enhance functionality.

The earliest emergence of phage-resistant bacteria in PaGZ-1-Aiia-treated cultures (Figure 4B) can be interpreted as a preliminary sign of potential resistance development, a critical factor to address in future therapeutic applications. We speculate that the *Aiia* lactonase itself might contribute to this dynamic through several possible avenues: for example, by generating nutrients via AHL degradation that could benefit resistant clones (dos Reis et al., 2012; Tinh et al., 2007), or by modulating bacterial behavior (Augustine et al., 2010; Anandan and Vittal, 2019) in a way that favors proliferative over colonizing phenotypes. Importantly, these are plausible mechanistic hypotheses derived from the literature to explain our observation, not confirmed findings of this study. If confirmed in future work, such interactions would highlight the need to design engineered phages with not only potent activity but also a consideration of how ancillary functions might shape long-term efficacy and resistance landscapes.

This study confirmed *in vitro* that both *Aiia* lactonase and *DP* depolymerase inhibit *P. aeruginosa* biofilm formation and disrupt mature biofilms (Figure 1B). These findings align with previous reports: Plasmid-borne *Aiia* expression in PAO1 yields less dense, architecturally compromised biofilms (Wang et al., 2007); Quorum Quenching lactonases Y2-Aiia attenuates virulence and biofilm formation in clinical isolate MMA83 (Malešević et al., 2020); Aiia_QSI-1_ inhibits biofilms in *A. hydrophila* (Zhang et al., 2019). Crucially, our work extends these observations by demonstrating that phage-encoded *Aiia* not only retains anti-biofilm activity but significantly enhances biofilm eradication compared to protein treatment alone. Similarly, while the *DP* depolymerase from *Pseudomonas* phage IME180 degrades exopolysaccharides and inhibits biofilms in strain Pa.1193 (Mi et al., 2019), we establish its broad-spectrum functionality against PAO1 biofilms.

Furthermore, the moderate enhancement in antibiofilm efficacy observed for PaGZ-1-Aiia and PaGZ-1-DP - evidenced by increased bacterial lethality within biofilms (Figure 5)—confirms successful functionalization through genome editing, albeit with incremental improvement. While our construct was successfully inserted, tolerated, and expressed, it is not yet optimized, there was no statistically significant difference in the disruption of mature biofilms between PAGZ-1-DP and the wild-type phage. We noted that the amplification fold of the DP gene in qPCR is lower than that of the *AiiA* gene (8,621 vs. 3,278, Figure 3C). This may indicate that the extended coding sequence of the *DP* gene (1,389 bp vs. *Aiia’s* 747 bp) imposes translational inefficiency or post-translational bottlenecks, ultimately limiting its potentiation of phage-mediated biofilm degradation.

Future iterations of this construct could enhance translational efficiency by optimizing insertion loci or incorporating N-terminal peptide extensions (Tats et al., 2006). The selection of *Aiia* or *DP* as candidate genes may represent a suboptimal strategy; identifying master regulators of biofilm formation for phage integration might yield superior efficacy. While the primary objective remains biofilm reduction, subsequent validation should encompass diverse drug-resistant clinical isolates. Systematic optimization of expression elements—including promoters, terminators, codon bias, gene copy number, and therapeutic transgenes (Lemon et al., 2019) —will advance development of high-potency engineered phages.

Collectively, this study establishes proof-of-concept for CRISPR-Cas9-mediated engineering of non-model *P. aeruginosa* phages, demonstrating their enhanced anti-biofilm and bactericidal efficacy *in vitro*. The developed platform paves the way for future therapeutic exploration and holds promise as a foundational strategy in the fight against multidrug-resistant *P. aeruginosa* infections.

## Data Availability

The data presented in this study are publicly available. The data can be found at: https://www.ncbi.nlm.nih.gov/genbank, accession numbers MH791399, MN227141 and MF788075.

## Footnotes

1
pubmlst.org/

## Supplementary material

SUPPLEMENTARY FIGURE S1 Schematic representations of the PGEX-6P-3-Aiia and PGEX-6P-3-DP plasmid vectors.

SUPPLEMENTARY FIGURE S2 Translucent plaques with halos formed by wild-type and engineered phages on PAO1 after 24–72 h incubation.
