# Supplementary figures and images for: Engineered Pseudomonas aeruginosa phages with quorum-quenching enzyme or depolymerase for inhibition of biofilm formation
Source: Front Microbiol. 2026 Jan 13;16:1752980. doi: 10.3389/fmicb.2025.1752980 (PMC12845329; doi:10.3389/fmicb.2025.1752980)

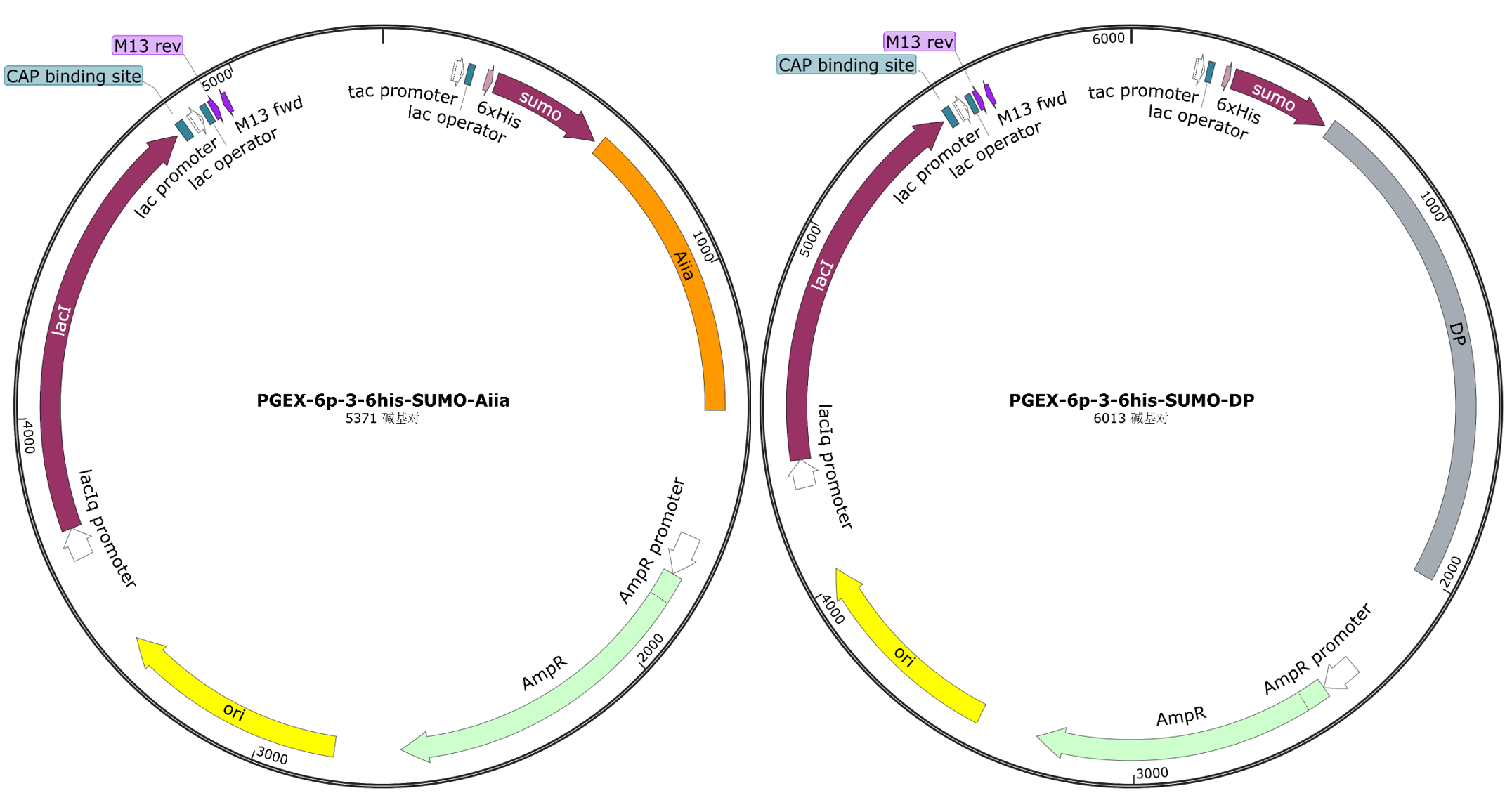

Supplement: SUPPLEMENTARY FIGURE S1 — Schematic representations of the PGEX-6P-3-Aiia and PGEX-6P-3-DP plasmid vectors. [file Image_1.tif]

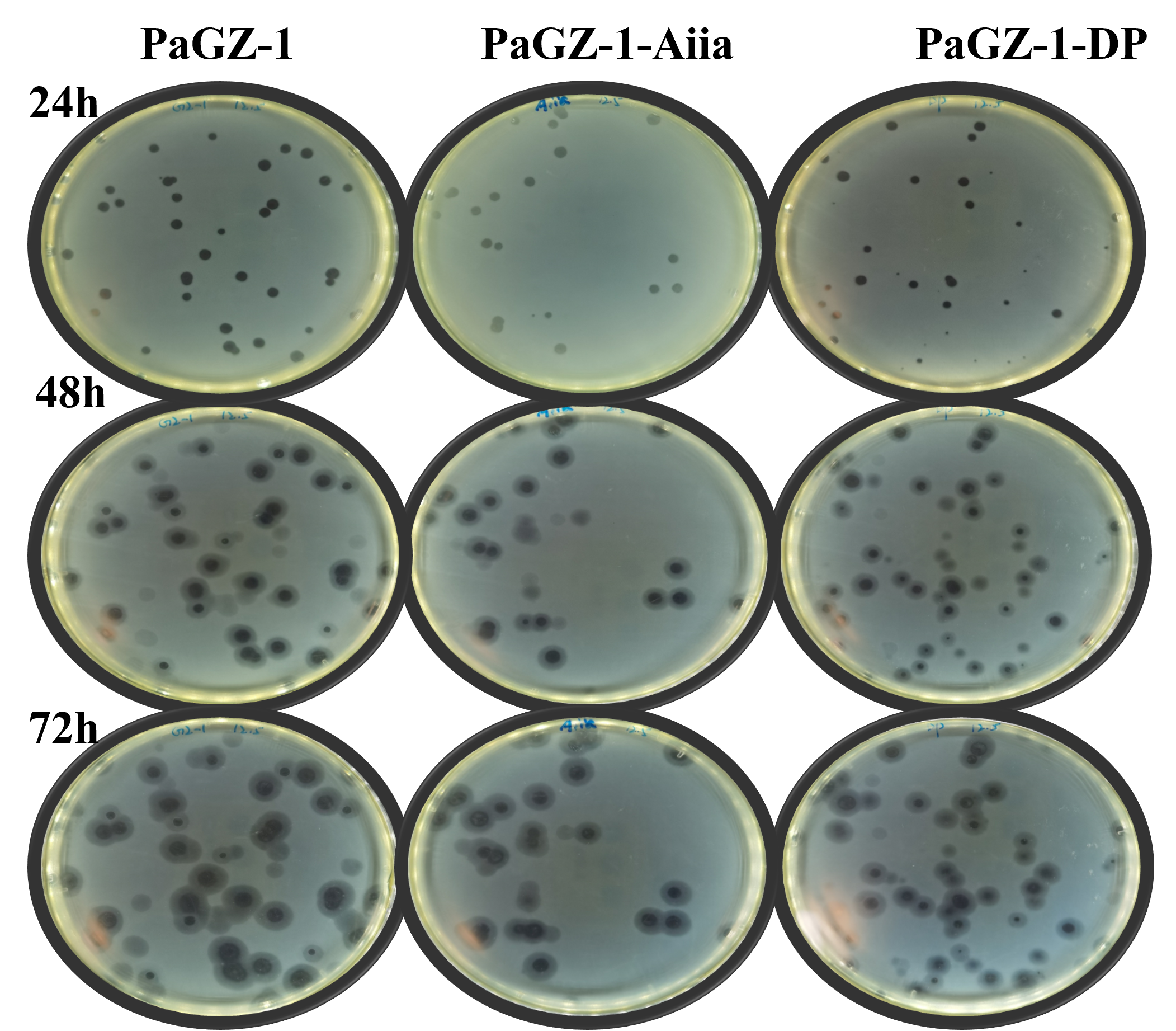

Supplement: SUPPLEMENTARY FIGURE S2 — Translucent plaques with halos formed by wild-type and engineered phages on PAO1 after 24–72 h incubation. [file Image_2.tif]
