# Supplementary material for: Engineered Pseudomonas aeruginosa phages with quorum-quenching enzyme or depolymerase for inhibition of biofilm formation
Source: Front Microbiol. 2026 Jan 13;16:1752980. doi: 10.3389/fmicb.2025.1752980 (PMC12845329; doi:10.3389/fmicb.2025.1752980)
Supplement: Supplementary file 3 [file Table_1.docx]

Supplement Table 1. Primers used in this study

| **Primers names** | **Sequence (5’-3’)** |
| --- | --- |
| PGEX-Aiia-F | AGGAGGTGGTGGATCAACCGTCAAGAAGCTGTACTTCG |
| PGEX-Aiia-R | AACTGCGGGTGGCTCCAGATGTATTCCGGGAATACTTTGCAGC |
| PGEX-Vector-F | TGGAGCCACCCGCAGTTC |
| PGEX-Vector-R | TTGATCCACCACCTCCTCCAC |
| PGEX-DP-F | AGGAGGTGGTGGATCAAGTACGTTGAGAGTAGACACTCTACAA |
| PGEX-DP-R | AACTGCGGGTGGCTCCAACGGGTCATATAGGAGAACGAG |
| promoter J23100 | TAGCTCAGTCCTAGGTACAGTGCTAGC |
| 1-insert-F | CCATAACTGGCAGAAGGCGA |
| 1-insert- R | GAAGCAAGTGGCACACCCGAT |
| 2-insert- F | CGAGCGCCTGTTAGGTAAG |
| mRFP1-F | ATGGCTTCCTCCGAAGACGTTA |
| 2-insert-R | CAGGTACAAAAACGCCCGTC |
